# Bidirectional Modulation of Beam Traversal Performance by Acetylcholine in the Cerebellar Nuclei

**DOI:** 10.1523/JNEUROSCI.1529-25.2026

**Published:** 2026-01-12

**Authors:** Cristiana I. Iosif, Robert A. R. Drake, Richard Apps, Zafar I. Bashir, Jasmine Pickford

**Affiliations:** ^1^University of Bristol, Bristol BS8 1TD, United Kingdom; ^2^King’s College London, London SE1 1UL, United Kingdom

**Keywords:** acetylcholine, balance, beam traversal, cerebellum, foot placement, motor performance

## Abstract

The cerebellum plays a key role in coordinating balance and movement control. Most studies of cerebellar function focus on the role of cerebellar glutamatergic inputs; thus, the roles of neuromodulatory inputs remain unexplored. We sought to determine whether the cholinergic projections from the Pedunculopontine tegmental nucleus (PPN) to the interpositus nuclei of the cerebellum are involved in modulating performance of a beam traversal task in rats. We manipulated cholinergic signaling in the cerebellum using chemogenetic and pharmacological methods. Experiments were conducted in male rats, except for studies specifically targeting PPN cholinergic neurons, which included rats of both sexes. Chemogenetic inhibition of either a mixed population of projections from the PPN to interpositus nuclei or specific inhibition of cholinergic PPN projections improved balance and foot placement on the beam traversal task. This effect is likely mediated by nicotinic receptors, as infusion of the nicotinic receptor antagonist mecamylamine improved balance and foot placement accuracy. In contrast, enhancing cholinergic signaling using the cholinesterase inhibitor physostigmine reduced accuracy of foot placement. Surprisingly, infusion of muscarinic receptor antagonists mimicked the effect of cholinesterase inhibition leading to impaired motor performance. We investigated the cellular effects of cholinergic receptor activation using adult rat cerebellar slices. Interpositus nuclear neurons exhibited decreased intrinsic excitability and reduced responsivity to synaptic inputs in the presence of a cholinergic agonist. Together, our findings indicate that low levels of acetylcholine in the cerebellar interpositus are optimal for performance on the beam traversal task, while enhancing cholinergic signaling decreases interpositus excitability and impairs task performance.

## Significance Statement

Pontine cholinergic centers are impacted in various disease states, with changes in cholinergic signaling linked to severe motor deficits such as in Parkinson’s disease. The cerebellum is central to the regulation of motor control, but the roles of acetylcholine (ACh) in cerebellar motor control are unclear. We set out to identify the function of the cholinergic pedunculopontine nuclei to the cerebellar nuclei pathway in the beam traversal task in rats and found that inhibiting this pathway improved motor performance. Our results suggest that low levels of ACh in the cerebellum are optimal for motor performance. The identified role of this pathway in normal motor behavior will reveal how this pathway may be targeted to improve motor symptoms in disease states.

## Introduction

Acetylcholine (ACh) plays a major role in modulating attention, cognition, and motor control (Hasselmo, 2006; Klinkenberg et al., 2011; Garcia-Rill et al., 2019). While its roles in attention and cognition through action in the forebrain are well described (Hasselmo, 2006; Klinkenberg et al., 2011; Huang et al., 2022), its central contributions to motor control through action in the cerebellum and hindbrain remain poorly understood. The pedunculopontine tegmental nucleus (PPN) is a major cholinergic center in the brainstem and is important for initiating movement and maintenance of gait (Mena-Segovia and Bolam, 2017; Garcia-Rill et al., 2019). Accordingly, loss of cholinergic neurons in the PPN is associated with impaired motor coordination in Parkinson’s disease (Tubert et al., 2019). Furthermore, deep brain stimulation of the PPN can improve motor function in humans (Lin et al., 2023), and specific activation of PPN cholinergic neurons can alleviate motor symptoms in a rat model of Parkinson’s disease (Pienaar et al., 2015).

We recently made a first step toward understanding the behavioral significance of ACh in the cerebellum by showing that infusion of muscarinic and nicotinic receptor antagonists targeting the interpositus nuclei of rats impaired motor performance but had no detectable effect on motor learning (Pickford et al., 2024). While our work demonstrated the role of ACh in cerebellar motor control, it gave no indication of the specific cholinergic pathway(s) involved. The same is true for other pharmacological studies assessing the effects of ACh receptor activation on neuronal activity, synaptic transmission, and plasticity (Rinaldo and Hansel, 2013; Pickford et al., 2019; Fore et al., 2020).

Cholinergic inputs from the PPN to the cerebellum are well positioned to impact cerebellar function in motor performance. The cerebellum receives cholinergic projections from several brainstem nuclei including the PPN, laterodorsal tegmental nucleus, lateral paragigantocellular nucleus, dorsal raphe nucleus, and vestibular nuclei (Woolf and Butcher, 1989; Jaarsma et al., 1997). As the PPN is known to be important for voluntary movements—in line with established roles of the intermediate cerebellum in coordinating balance, gait, and fine motor control (Ito, 1972; Manto et al., 2012; De Zeeuw and Ten Brinke, 2015)—we identified the PPN as a candidate region for mediating cerebellar control of motor behavior via ACh signaling in the interpositus nuclei. A functional connection between these two brain regions is supported by evidence that electrical stimulation of the PPN evokes short-latency excitatory responses in the cerebellar nuclei, which are dependent on ACh receptors (Vitale et al., 2016). The role of this pathway in normal motor behavior remains poorly understood, and therefore we set out to interrogate its role in habitual motor performance.

We performed cerebellar circuit manipulations in rats trained on a beam traversal task, testing their ability to place paws accurately during locomotion while maintaining balance on a narrow walkway. We present the unexpected finding that chemogenetically inhibiting the PPN input to the interpositus nuclei—by targeting a mixed population of neurons or cholinergic neurons specifically—improved balance and foot placement measures during traversal of a narrow beam in well-trained rats. Conversely, enhancing cholinergic signaling in the cerebellar interpositus via infusion of a cholinesterase inhibitor reduced foot placement accuracy. Together, our findings suggest that cerebellar modulation of skilled beam traversal is sensitive to too much or too little ACh—an important consideration for administration of cholinergic agents in a clinical setting.

## Materials and Methods

### Animals

Male Lister hooded rats (*n* = 60, Envigo) and male and female transgenic Long–Evans ChAT-Cre rats (*n* = 7, LE-Tg(Chat-Cre)5.1Deis, Rat Research and Resource Center, donor Karl Deisseroth) were pair-housed under a 12 h light/dark cycle (lights on 20:15–08:15), with all behavioral experiments conducted during the dark phase. During recovery from surgery, animals had *ad libitum* access to food and were then restricted to 16 g of standard chow per day for the duration of the behavioral study. Water was available *ad libitum*. A reward of 45 mg purified rodent tablets was used during behavioral testing (TestDiet LabTab AIN-76, 5TUL, catalog #1811155).

### Surgical procedures

Rats were anesthetized with ketamine/medetomidine (50/0.3 mg/kg initial dose, i.p., with supplementary doses as needed) and positioned in a stereotaxic frame. A heated blanket maintained body temperature at ∼37°C. For chemogenetic manipulations, viral vectors were injected bilaterally into the PPN at 7.8 mm posterior and 1.9 mm lateral relative to the bregma, at a depth of 6.5 mm from the dura. For the chemogenetic inhibition of PPN–cerebellar input experiments, the hM4Di group received AAV5-hSyn-hM4D(Gi)-mCherry (250 nl, 8.6 × 10^12^ vg/ml), and the control group received AAV5-hSyn-EGFP (250 nl,1.2 × 10^12^ vg/ml). For cholinergic targeting, ChAT-Cre hM4Di rats were injected with AAV2-hSyn-DIO-hM4D(Gi)-mCherry (350 nl, 1 × 10^12^ vg/ml), and controls were injected with AAV2-EF1a-DIO-mCherry (350 nl, 1 × 10^12^ vg/ml). Viral vectors were injected at 200 nl/min, with a 3 min dwell time.

A bilateral guide cannula (26 G, 5 mm intercannular distance) targeting the cerebellar interpositus nuclei was implanted at 11.16 mm posterior, 2.5 mm lateral, and 4.9 mm deep relative to the bregma. The cannula was secured to the skull with skull screws and dental cement, and dummy cannulae were inserted to maintain patency between infusions. Post-surgery, animals were given Metacam (1 mg/kg, s.c.) and saline (0.9%, 10 ml/kg, s.c.) and were regularly monitored until they regained pre-surgery weight.

### Infusions

Clozapine-*N*-oxide (CNO) was dissolved in DMSO at 3 mM and then diluted to 3 µM in 0.9% saline. For pharmacological experiments, mecamylamine (nicotinic antagonist) was prepared at 50 µM in saline; VU0255035 (M1 antagonist) was dissolved in DMSO to 20 µM and then diluted to 1 µM in saline; AF-DX116 (M2 antagonist) was dissolved to 1 M in HCl, neutralized with NaOH, and diluted to 2.5 mM in saline; 4-DAMP (M3 antagonist) was prepared at 4.65 mM in saline at 37°C; and physostigmine (cholinesterase inhibitor) was diluted to 14.5 mM in saline. Vehicle infusions were 0.9% saline for mecamylamine and physostigmine and 0.1% DMSO in saline for chemogenetic and muscarinic experiments. The data obtained with physostigmine infusion were also included in a previous study, where it was analyzed using different parameters [traversal time and total number of complete foot slips in Pickford et al. (2024); graded foot slips (scored on a scale from 1 to 3), tail movements (novel measure) and traversal time in current study].

Infusions were counterbalanced, and the experimenter was blinded to the identity of each infusion. Bilateral infusion needles (33 G, 1 mm protrusion below guide cannula) were inserted into the guide cannulae, and 500 nl of solution was delivered over 1.5 min into each side of the cerebellum. Cannulae were left in situ for 3 min before dummy cannulae were replaced. Behavioral testing began 15 min after the start of the infusion.

### Beam traversal

**Figure 1.**
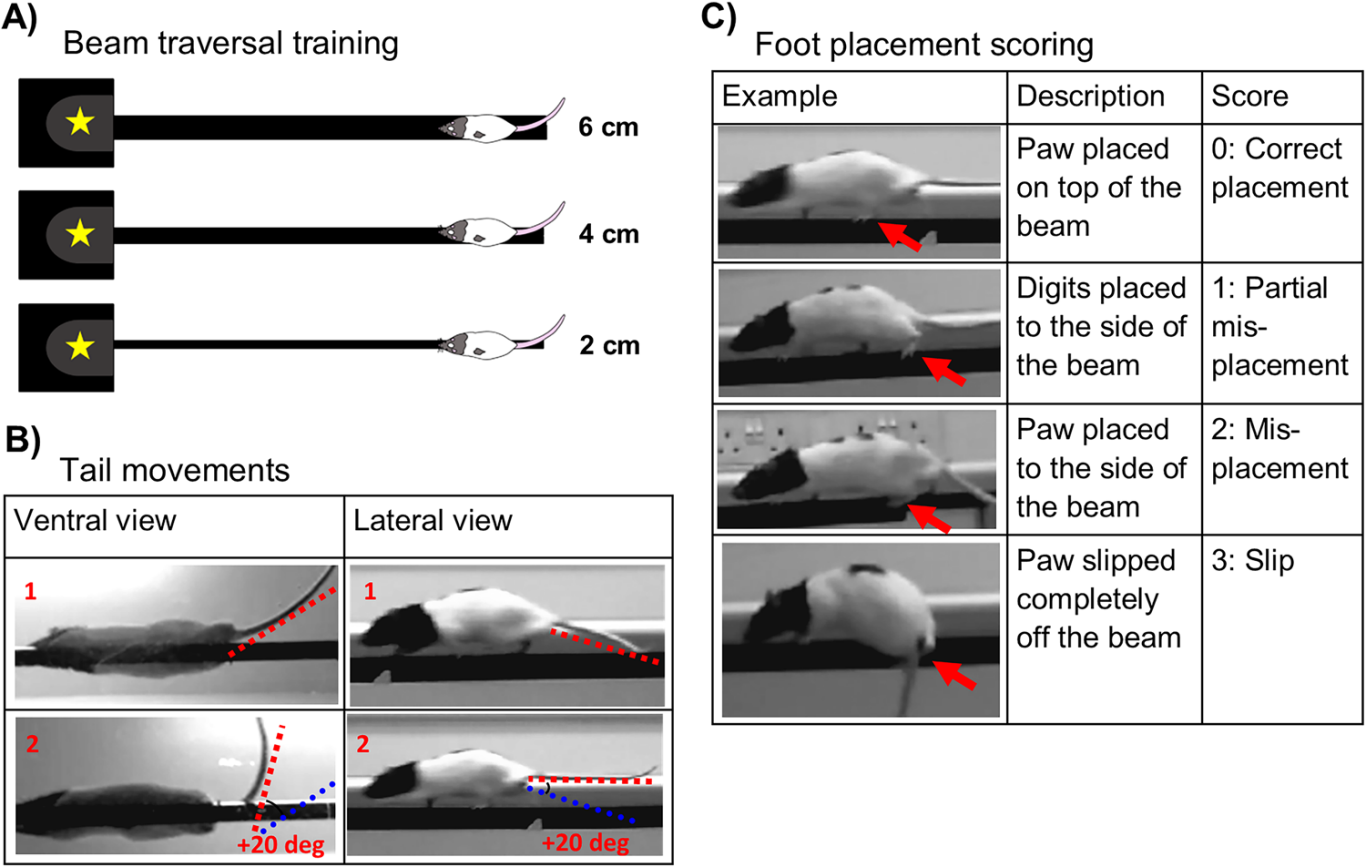
Beam traversal task and scoring. ***A***, Rats were trained to traverse a narrow beam to obtain a reward. They were trained on a 6-cm-wide beam followed by a 4-cm-wide beam (3 sessions of 6 trials each per width) and then on a 2-cm-wide beam (4 sessions of 6 trials each). Testing was carried out using the 2 cm beam. ***B***, Tail movement scored from the ventral view as a lateral displacement of the tail and from the lateral view as a vertical displacement of the tail. For both panel views, 1 indicates the initial tail position and 2 shows the final tail position after movement, a radial position change of at least 20°. ***C***, Foot placement score graded in order of magnitude.

Rats were trained to traverse a narrow beam. The beam was 164 cm long, supported at both ends by 92 cm high poles. The start of the beam was exposed, and a dark opaque plastic box was at the target end (Pickford et al., 2024). Rats were habituated to the beam and target box, where during training and testing five reward pellets were placed between each trial to encourage traversal. Training began on a 6-cm-wide beam (six trials for three sessions), followed by a 4-cm-wide beam (six trials for three sessions) and then a 2-cm-wide beam (six trials for four sessions; Fig. 1*A*). With training, animals learned to consistently place their paws on top of the beam. On test day, rats completed four baseline trials, received an infusion of the compound of interest into the cerebellar nuclei, and then performed two test trials (averaged to obtain performance per treatment). A trial began when the rat started traversing the beam and ended when it entered the end box with all paws.

**Figure 2.**
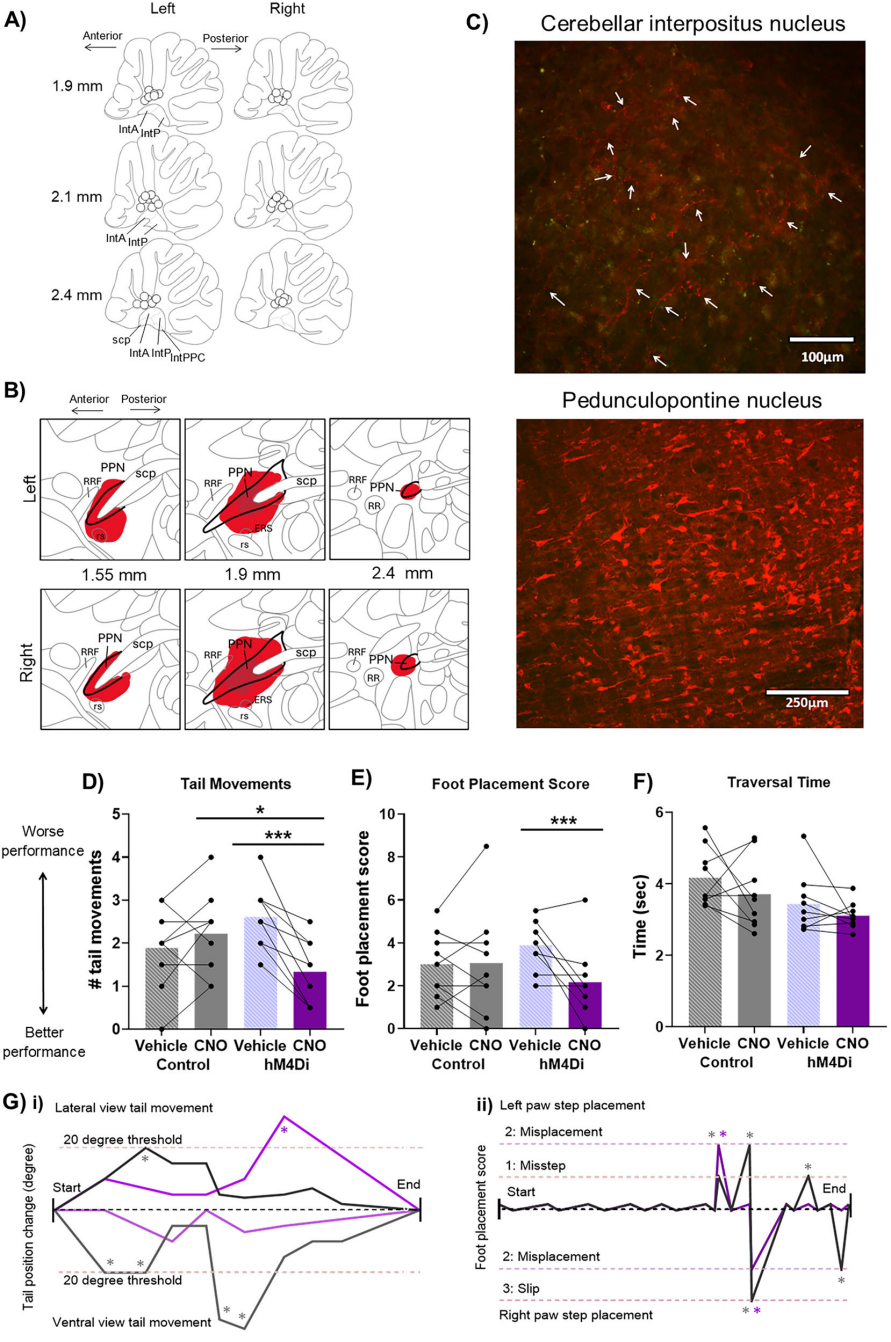
Chemogenetic inhibition of PPN input to cerebellar nuclei improved motor performance. ***A***, Cannulae implant location for *n* = 18 rats involved in the study (*n* = 9 hM4Di-expressing and *n* = 9 control). Mapping done in sagittal plane; measures indicate distance from midline. IntA, interpositus anterior; IntP, interpositus posterior; IntPPC, interpositus posterior parvicellular part; scp, superior cerebellar peduncle. ***B***, Distribution of cells expressing hM4Di across the PPN. Mapping done in sagittal plane and measurements indicate distance from midline. Red marking indicates viral spread across animals, and dark red corresponds to the center of the viral injection, the area transfected across all animals. PPN, pedunculopontine tegmental nucleus; scp, superior cerebellar peduncle; rs, rubrospinal tract; RRF, retrorubral field; ERS, epi-rubrospinal nucleus; RR, retrorubral nucleus. ***C***, Viral vector injection in the PPN led to hM4Di-mCherry expression in PPN neurons. hM4Di-mCherry–expressing fibers were identified across the anterior and posterior interpositus nuclei and parts of the lateral nuclei. Scale bar: PPN, 250 µm; cerebellar interpositus nuclei, 100 µm. Arrows indicate labeled fibers. ***D–F***, Activation of hM4Di receptors on PPN terminals in the cerebellum (via CNO infusion) during the beam traversal task resulted in a significant reduction of (***D***) tail movements and (***E***) foot placement score, with no change in (***F***) traversal time. Statistical analysis tested for interactions between group and treatment and post hoc simple effects analysis for each treatment and each group; stars represent statistical significance: *p < 0.05; **p < 0.01; ***p < 0.001. ***G***, Example demonstrating the quantification of scored behavior in an example trial. Data example taken from a hM4Di animal with vehicle infusion (gray traces) and CNO infusion (purple traces). ***i***, Tail movement quantification for ventral and lateral view: any change >20° from the previous position was classified as a balance adjustment tail movement (indicated by gray stars for vehicle infusion and purple stars for CNO infusion). ***ii***, Foot placement score across the trials showing both the left and right paw step placement: scores of each foot placement from the start of the trial to the end; gray stars for vehicle infusion and purple stars for CNO infusion indicate the occurrence of missteps (partial misplacement), misplacements, and foot slips.

**Figure 3.**
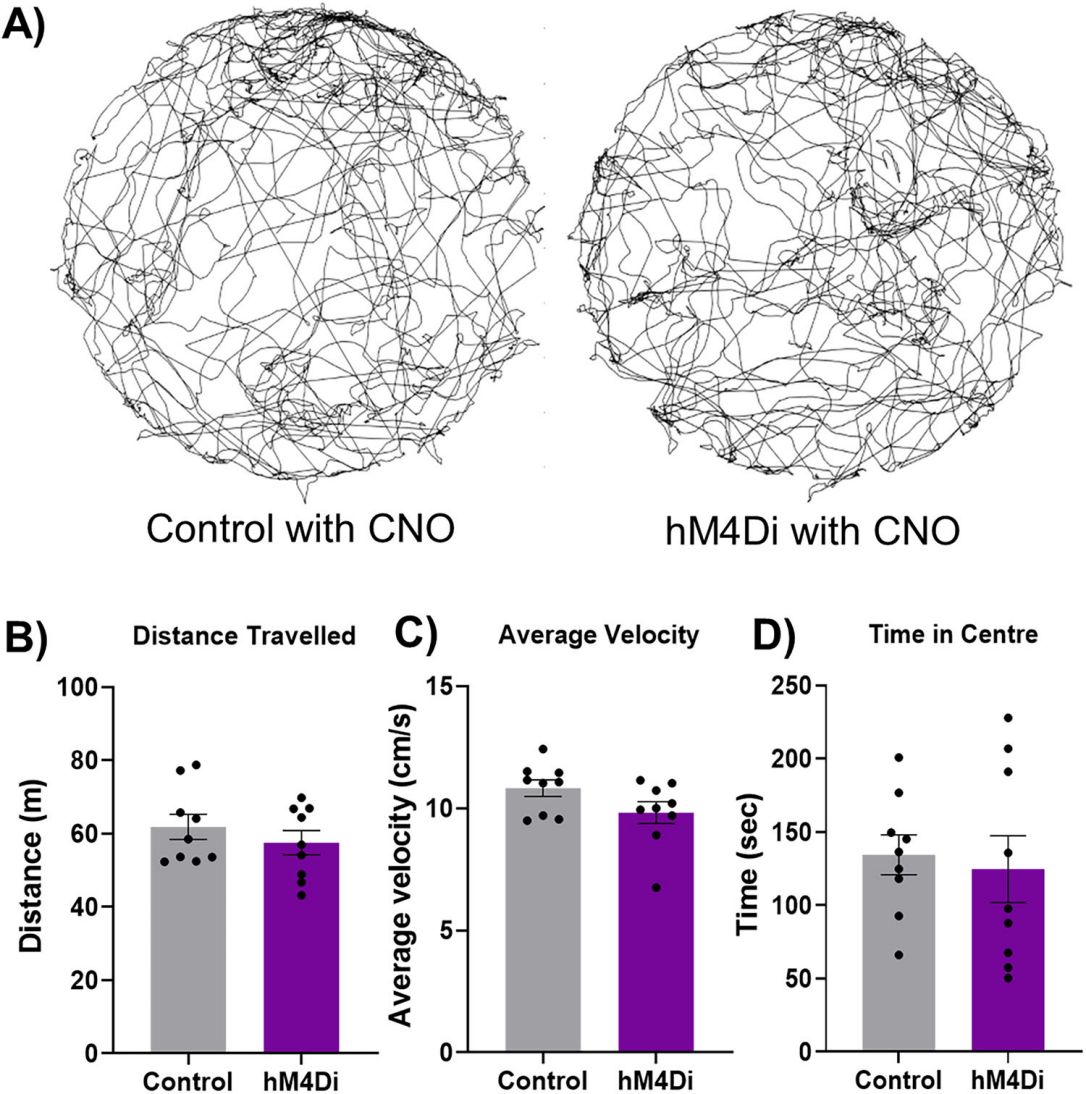
Open-field exploration following chemogenetic inhibition of PPN input. ***A***, Example trajectories of a control rat (left) and rat expressing hM4Di in PPN cells (right) following cerebellar CNO infusion. ***B–D***, Manipulating the PPN input to the cerebellar nuclei via CNO infusion into the cerebellum of hM4Di-expressing rats did not affect the (***B***) total distance traveled, (***C***) average velocity, or (***D***) time spent exploring the center, relative to control conditions (*n* = 9 per group).

Task performance was recorded using three cameras recording at 30 fps: one lateral and two ventral to the beam. The experimenter was blind to the manipulation when scoring the videos using BORIS (Friard and Gamba, 2016, v 8.27.7). Task performance was assessed by (1) hindpaw placement score (lower scores indicating better coordination; Gholamzadeh et al., 2021; Fig. 1*B*), (2) the number of tail movements (reduction indicating improved performance; Wecker et al., 2013; Lacava et al., 2024; Fig. 1*C*), and (3) traversal time (reduced time indicating improved motor performance; Luong et al., 2011).

Foot placement errors were categorized, for every placement, as correct placement (Score 0, digits placed on top of the beam), partial misplacement (Score 1, digits placed to the side of the beam), misplacement (Score 2, foot placed to the side of the beam), or slip (Score 3, foot slipped off the beam) as shown in Fig. 1*C*. This produces a total foot placement score equaling the sum of all foot placement scores. Tail movements help to maintain stability of the trunk during locomotion, either passively (acting as a counterweight to lower the center of mass) or actively (making compensatory movements to account for perturbations or stepping errors; Lacava et al., 2024). Tail movements were recorded as any radial movement of over 20° originating at the base of the tail, up or down or to either side of the beam (Fig. 1*B*; Lacava et al., 2024).

### Open-field exploration tasks

Rats explored a 90-cm-diameter circular arena on a black plastic mat for 10 min. Movement was tracked using DeepLabCut, and total distance, average velocity, and time spent in the center (30 cm diameter) were calculated with MATLAB (2019b).

### Histological processing

Following behavioral testing, animals were deeply anesthetized with sodium pentobarbital and transcardially perfused with saline (0.9%) followed by 4% paraformaldehyde (PFA). Brains were dissected and stored in PFA for 24 h and then transferred to a cryoprotective solution [30% sucrose in 0.1 M phosphate buffer (PB)]. After embedding in gelatin, brains were freeze-sectioned at 40 µm using a Leica SM2000R microtome. For animals without viral injections, sections were mounted with gelatin, and implant locations were mapped using light microscopy. For animals with viral injections, immunohistochemistry was performed to visualize protein expression.

hM4Di-expressing wild–type and ChAT-Cre animals were stained for mCherry using rabbit anti-mCherry (Biovision 5993-100) primary antibody (1:2,000 in phosphate-buffered saline with Tween and normal horse serum, PBS-T + NHS) and donkey anti-rabbit Alexa Fluor 594 (R37119) secondary antibody (1:1,000 in PB). Control animals were stained for GFP with chicken anti-GFP (ab13970) primary antibody (1:5,000 in PBS-T + NHS) and donkey anti-chicken Alexa Fluor 488 (A78948) secondary antibody (1:1,000 in PB). The tissue from ChAT-Cre rats was co-immunostained for choline acetyltransferase (ChAT) using goat anti-ChAT (AB144P) primary antibody (1:500 in PBS-T + NHS) and donkey anti-goat Alexa Fluor 488 (A32814) secondary antibody (1:1,000 in PB). Sections were mounted with gelatin and visualized with a Zeiss Axioskop microscope using two filter sets: (1) ET-GFP/Alexa Fluor 488 (excitation 470/40 nm, emission 525/50 nm) and (2) ET-mCherry/Alexa Fluor 594 (excitation 560/40 nm, emission 630/75 nm). Cell counting for ChAT-Cre samples was performed in 500 × 500 µm square grids in 3× single-plane images per hemisphere, and only neurons with complete soma and nuclear outline were included in the classification. Images were captured using the Ocular software.

### Slice preparation for in vitro electrophysiology

Rats were deeply anesthetized with isoflurane, and the brain was dissected and placed in warm (34°C) oxygenated sucrose solution (in mM: 189 sucrose, 10 d-glucose, 26 NaHCO_3_, 3 KCl, 5 MgSO_4_·7H_2_O, 1.25 NaH_2_PO_4_, 0.2 CaCl_2_·2H_2_O). Coronal cerebellar slices (300 µm) were cut using a Campden Vibrotome (Model 7000smz-2) with a ceramic blade, vertical vibration <0.4 µm, and horizontal vibration 1 mm at 90 Hz, with 0.01 mm/s slicing speed. The slicing chamber was maintained at 34°C (±0.7°C) using a Campden Temperature Controlled Warming Tissue Bath (Model 7611A).

Slices were recovered in artificial cerebrospinal fluid (in mM: 125 NaCl, 2.5 KCl, 25 d-glucose, 25 NaHCO_3_, 1.25 NaH_2_PO_4_, 1 MgCl_2_, 2 CaCl_2_) at 34°C for 1 h and then held at room temperature until used for experiments.

### Whole-cell electrophysiological recordings

Whole-cell patch–clamp experiments were conducted in a recording chamber maintained at 28–30°C. Cells were visualized with a Nikon Eclipse E600 FN microscope with differential interference contrast optics and a 40× objective. A MultiClamp 700B amplifier (Molecular Devices) and WinLTP acquisition software (Anderson et al., 2012) were used for data collection. Whole-cell recordings were performed with glass pipettes pulled from borosilicate capillaries (GC150F-10, Harvard Apparatus) using a PC-100 Narishige Micropipette Puller.

Intrinsic activity was recorded in current-clamp mode with a K-gluconate internal solution containing biocytin (in mM: 120 K-gluconate, 10 KCl, 33.3 HEPES, 0.5 EGTA, 0.3 GTP-Na, 2 ATP-Mg, 1 MgCl_2_, 2 NaCl, 6.7 biocytin). The experimental protocol consisted of a 10 min baseline period, followed by 10 min bath perfusion of 10 µM carbachol (diluted from a 100 mM stock in ddH_2_O and stored at −18°C). Cellular activity was recorded continuously at a 40 kHz sampling rate. The spontaneous firing rate, extracted from the final 3 min of both baseline and carbachol perfusion, was determined using a 0 mV threshold for peak detection in MATLAB (2019b). Action potential (AP) waveforms were extracted 2 ms before and after detected peaks, with AP threshold defined as a 5 mV increase per 0.5 ms; AP threshold, half-width, rate of rise, and after-hyperpolarization peak (AHP) were calculated.

Intrinsic membrane properties were assessed by injecting a small holding current (±13 pA) to stabilize the membrane potential (Vm) between −49 and −54 mV and a square −300 pA hyperpolarizing current. Input resistance (Ri = Vm/current), membrane time constant (tau), and sag (percentage change between the final 10 ms of steady-state hyperpolarization and the predicted Vm without sag) were analyzed in response to hyperpolarizing current injection using MATLAB (2019b). For local stimulation experiments, a cesium-based internal solution was used (in mM: 130 CsMeSO_4_, 10 HEPES, 0.5 EGTA, 4 GTP-Na, 0.3 ATP-Mg, 2 QX-314-Cl-, 4 KCl, 3.7 biocytin). Cells were held at a membrane potential of −54 mV in voltage clamp, and a stimulating electrode was placed in the white matter adjacent to the interpositus cerebellar nuclei. Single- or paired-pulse (100 ms interval) stimulation was applied every 30 s (Pickford et al., 2019). The amplitude of inhibitory postsynaptic currents (IPSCs; isolated with AMPA and NMDA receptor antagonists NBQX, 5 µM and D-AP5, 50 µM) and excitatory postsynaptic currents (EPSCs; isolated with GABAergic receptor antagonist picrotoxin, 50 µM) were recorded. Amplitudes of postsynaptic currents at 10 min of baseline and carbachol perfusion were compared, with series resistance maintained at 10–20 MΩ throughout.

### Tissue processing for cell morphology

Post-recording, slices were preserved in 4% PFA and stained for biocytin using the ABC reagent (Vector Labs, VECTASTAIN ABC AP Kits), followed by DAB staining with Nickel (Vector Labs, DAB Substrate Kit, Peroxidase HRP). After staining, slices were dry-mounted, cleared with histoclear, and coverslipped with DPX mountant glue. Stained cells were visualized in bright field using a Zeiss Axioskop. Neurite ramifications were mapped using GIMP (v 2.10.30), and soma diameter and neurite ramification were measured in ImageJ.

### Experimental design and statistical analysis

Statistical analyses were conducted in R (v 4.4.1), and graphs were created using GraphPad Prism (v10) as bar charts displaying the mean and individual data points.

All behavioral experiments involved infusion of active agents (CNO or drug) and vehicle; in the chemogenetic experiments where the animals were divided into an active group expressing hM4Di and a control group expressing a fluorophore, vehicle and CNO infusions were conducted in both groups. Infusions in all rats were counterbalanced, and the experimenter was blinded to the agents infused during the conduction of the experiment and during analysis. Animals received vehicle infusion for each experiment conducted, and overall performance did not differ across all experimental conditions (Fig. S1; *χ*^2^_(4)_ = 2.1; *p* = 0.7 for tail movements; *χ*^2^_(4)_ = 7.8; *p* = 0.1 for foot placement score, analyzed based on group factor as detailed below).

For the behavioral data statistical analysis, we fitted our data with a generalized linear model (GLM) in the following structure (with variations depending on the experimental design; Vaccari et al., 2021):

GLM(DependentVariable∼AnimalID+Group+Treatment+Treatment*Group).

The key feature of the model is that it accounts for multiple comparisons and individual variability while assessing (1) the effect of treatment (treatment factor assesses whether the different treatment led to different effects in the population); (2) the role of the different animal groups (whether the different groups behaved differently); and (3) the interactions between treatment and groups (assessing whether the effect of treatment was different within the different groups).

Generally, all behavioral experiments were analyzed using GLMs (using R); however, the type of GLMs used differed depending on the type of data collected. For all beam traversal task experiments, discreet variables (count data) from tail movements and foot placement scores were analyzed with Poisson regression GLMs. In these cases, to quantify the importance of each factor (treatment, group, or treatment–group interactions), the goodness of fit of the models was assessed using a likelihood ratio *χ*^2^ test (Dominicus et al., 2006). Where appropriate, post hoc simple effects analysis quantified the differences between the treatment factors within each group. Continuous variables such as beam traversal time and open-field measurements were analyzed using GLMs assuming Gaussian distribution of data followed by analysis of variance (ANOVA) comparison; where appropriate, a multiple-comparison test was performed to account for differences between the treatment factor within each group.

Behavioral data obtained from the beam traversal task following chemogenetic manipulation of PPN input to the cerebellar nuclei were analyzed using GLMs to examine interactions between the treatment factor (CNO infusions or vehicle) and the group factor (hM4Di or control fluorophore). Post hoc simple effect analyses assessed the treatment effect within each group and group differences under the same treatment. Beam traversal time in the hM4Di experiment were analyzed using a GLM with Gaussian distribution and ANOVA with Tukey’s test for multiple comparisons. Chemogenetic open-field data (comparing open-field exploration between hM4Di and control groups with CNO) was analyzed with GLMs assessing treatment effects on performance. We used 18 male Lister hooded rats for the chemogenetic inhibition of PPN projection to the cerebellar nuclei (nine rats expressing hM4Di and nine rats expressing a control fluorophore). For the cholinergic-specific chemogenetic inhibition experiment, we used seven male and female Long–Evans ChAT-Cre rats [four ChAT-Cre rats expressing hM4Di (two males and two females) and three ChAT-Cre rats expressing a control fluorophore (two females and one male)].

Pharmacology behavior data comparing beam traversal performance between infusion of different pharmacological agents and saline vehicle were analyzed with GLMs assessing treatment effects on performance. Pharmacology experiments using cholinergic agonist physostigmine and nicotinic receptor antagonist mecamylamine were performed in nine male Lister hooded rats. The muscarinic receptor antagonist experiments were conducted in a separate group of six male Lister hooded rats, and multiple pharmacological agents were infused alongside vehicle in a counterbalanced manner. Post hoc simple effects analysis compared the effect of each treatment with vehicle; for traversal time, a Bonferroni correction was used to account for multiple comparisons.

Electrophysiological data were obtained from the same cells at baseline and following pharmacological activation of cholinergic receptors using the agonist carbachol. We compared baseline (low ACh) conditions with prolonged activation of cholinergic receptors (10 min agonist application). For membrane potential and spontaneous firing rate, we also compared baseline conditions with early activation of cholinergic receptors (min 1–3 agonist application). The data were analyzed using parametric paired *t* tests which account for repeated measures. In the current study, we recorded the properties of 30 interpositus nuclear neurons from slices obtained from 30 male Lister hooded rats (one cell recording per animal)—this was due to the difficulty of obtaining viable adult rat cerebellar nuclear slices (Huang and Uusisaari, 2013).

## Results

### Effects of chemogenetic inhibition of PPN input to cerebellar nuclei

We first asked how chemogenetic inhibition of the PPN projection to the cerebellar nuclei affects beam traversal performance. We transduced the PPN with the inhibitory hM4Di receptor (or with green fluorescent protein, GFP, in a control group) and implanted a bilateral guide cannulae over the interpositus nuclei to deliver either CNO or vehicle (Fig. 2*A* shows the cannula placement for *n* = 18 rats in both groups). Expression of hM4Di receptors was apparent in PPN neurons in *n* = 9 rats, in both its caudal subdivision (rich in cholinergic neurons) and its rostral subdivision (rich in GABAergic neurons; Fig. 2*B*,*C*). In the cerebellum, hM4Di-expressing fibers were identified in the interpositus cerebellar nuclei, distributed across both its anterior and posterior divisions (Fig. 2*C*), as well as in the lateral nuclei. GFP expression was comparable in the control group.

Rats expressing hM4Di or GFP were trained to traverse a narrow beam to obtain sugar pellet rewards and then infused with CNO or vehicle prior to testing on the beam (Figs. 1, 2*D*). For each trial, we scored foot placement and tail movements (Fig. 2*G*), and the final results are presented as average across two trials. A GLM considered interactions between treatment and group and found statistically significant differences between the effect of CNO and vehicle on the animals expressing hM4Di receptors compared with the control animals expressing GFP for number of tail movements (*χ*^2^_(1)_ = 11.9; *p* = 0.0005; Fig. 2*D*) and foot placement score (*χ*^2^_(1)_ = 9.6; *p* = 0.001; Fig. 2*E*). Infusion of CNO significantly reduced the number of tail movements in rats expressing hM4Di compared with vehicle infusion (post hoc simple effects analysis for treatment factor in the hM4Di group; *χ*^2^_(1)_ = 14.4; *p* = 0.0001). The number of tail movements in hM4Di rats following CNO infusion was also significantly lower than the following CNO infusion in control rats (post hoc simple effects analysis for the group factor with CNO treatment; *χ*^2^_(1)_ = 5.3; *p* = 0.02). We identified no statistically significant difference within the control group, between CNO infusion and vehicle (post hoc simple effects analysis for treatment factor in the control group; *χ*^2^_(1)_ = 0.98; *p* = 0.32). As well as this, CNO infusion decreased foot placement score compared with vehicle in the hM4Di group (treatment factor post hoc simple effects analysis; *χ*^2^_(1)_ = 17.3; *p* = 0.0003) while having no statistically significant effect on the control group (*χ*^2^_(1)_ = 0.01; *p* = 0.89). In contrast, no effect of the CNO treatment on hM4Di activation was detected on traversal time (*F*_(1, 32)_ = 0.05; *p* = 0.82; Fig. 2*F*). These data demonstrate that disrupting PPN input to the cerebellar nuclei input leads to improved balance and limb coordination as shown by decreased tail movements and foot placement scores during a beam traversal task.

To assess whether changes in motor performance following chemogenetic inhibition of PPN input to the cerebellar nuclei were specific to performance of beam traversal (a skilled motor task) or motor control more generally, CNO infusions were performed in the control and hM4Di groups while rats explored an open-field arena (Fig. 3*A*). We found no statistically significant difference in the total distance traveled in the arena (*t*_(16)_ = 0.9; *p* = 0.38; Fig. 3*B*), the average velocity of movement (*t*_(16)_ = 1.7; *p* = 0.09; Fig. 3*C*), nor the average time spent in the center (*t*_(16)_ = 0.36; *p* = 0.71; Fig. 3*D*). These results show that inhibiting the PPN input to the cerebellar nuclei does not affect general locomotion but may be engaged in a task-dependent manner. However, this does not exclude the possibility that the PPN input to cerebellar nuclei also plays a role in other types of behavior not studied in the present experiments.

### Effects of inhibiting the cholinergic PPN projection to the cerebellar nuclei

**Figure 4.**
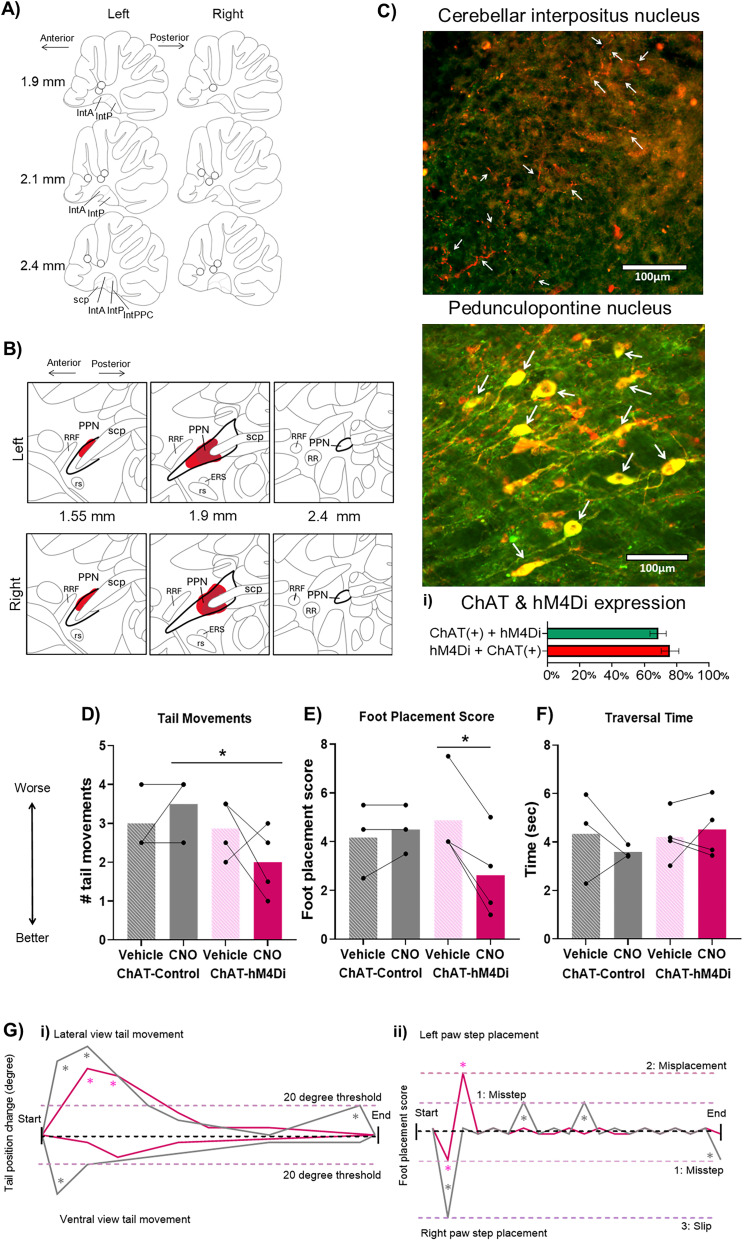
Inhibiting the cholinergic PPN projection to the cerebellar nuclei improved motor performance. ***A***, Cannulae implant location for *n* = 7 rats involved in the study (*n* = 4 hM4Di-expressing in ChAT-Cre rats and *n* = 3 control rats). Mapping done in sagittal plane; measures indicate distance from midline. IntA, interpositus anterior; IntP, interpositus posterior; IntPPC, interpositus posterior parvicellular part; scp, superior cerebellar peduncle. ***B***, Distribution of cells expressing hM4Di across the PPN. Mapping done in sagittal plane; measures indicate distance from midline. Red marking indicates viral spread across animals, and dark red corresponds to the center of the viral injection, the area transfected across all animals. PPN, pedunculopontine tegmental nucleus; scp, superior cerebellar peduncle; rs, rubrospinal tract; RRF, retrorubral field; ERS, epi-rubrospinal nucleus; RR, retrorubral nucleus. ***C***, In ChAT-Cre rats, Cre-dependent (DIO) viral vector injection in the PPN led to hM4Di-mCherry expression in PPN neurons. Colabeling was identified based on mCherry (red) and ChAT (green) overlap (yellow) in a single-plane image. In pedunculopontine nucleus image, arrows point to yellow-signal cells identified as colabeled; in interpositus nucleus, image arrows point to labeled fibers. Scale bars, 100 µm. ***i***, Coimmunohistochemical labeling for hM4Di-mCherry and ChAT in the PPN identified that the majority of the cells were colabeled—68.5% of all PPN ChAT-neurons also expressed hM4Di, and 75.9% of hM4Di-expressing neurons were ChAT-positive. hM4Di-mCherry–expressing fibers were identified across the anterior and posterior interpositus nuclei and parts of the lateral nuclei. ***D–F***, Activation of hM4Di receptors on cholinergic PPN terminals in the cerebellum via CNO infusion during the beam traversal task resulted in a significant reduction of (***D***) tail movements and (***E***) foot placement score, with no change in (***F***) traversal time. Statistical analysis tested for interactions between group and treatment and post hoc simple effects analysis for each treatment and each group; stars represent statistical significance: *p < 0.05. ***G***, Example demonstrating the quantification of scored behavior in an example trial. Data example taken from a ChAT-hM4Di animal with vehicle infusion (gray traces) and CNO infusion (magenta traces). ***i***, Tail movement quantification for ventral and lateral view: any change >20° from the previous position was classified as a balance adjustment tail movement (indicated by gray stars for vehicle infusion and magenta stars for CNO infusion). ***ii***, Foot placement score across the trials showing both the left and right paw step placement: scores of each foot placement from the start of the trial to the end; gray stars for vehicle infusion and magenta stars for CNO infusion indicate the occurrence of missteps (partial misplacement), misplacements, and foot slips.

The PPN contains cholinergic, glutamatergic, and GABAergic neurons (Spann and Grofova, 1992; Mena-Segovia and Bolam, 2017). Therefore, our next step was to specifically dissect the contribution of cholinergic PPN projections to cerebellar nuclei in beam traversal performance. This was done using Cre-dependent chemogenetic inhibition in *n* = 4 ChAT-Cre rats (Mantanona et al., 2019). The cannula placement for *n* = 7 ChAT-Cre rats (four ChAT-Cre rats expressing hM4Di and three control ChAT-Cre rats expressing mCherry) used in the study is shown in Figure 4*A*.

We identified the expression of hM4Di in the caudal PPN (Fig. 4*B*). To determine the specificity of hM4Di expression in cholinergic neurons in the PPN, we costained for mCherry (to indicate expression of hM4Di receptors) and ChAT (to identify cholinergic neurons) on PPN sections from injected ChAT-Cre rats. mCherry expression in the caudal PPN and was primarily colocalized with expression of ChAT (Fig. 4*C*). On average 73.8 ± 3 transfected cells/mm^2^ were identified in caudal PPN of *n* = 5 ChAT-Cre rats. We expected between 70 and 90% of mCherry cells to be ChAT-positive (Witten et al., 2011; Tryon et al., 2023; Yu et al., 2023). Approximately 76% of the cells expressing mCherry in the PPN were ChAT positive (Fig. 4*C*i), and similarly, ∼70% of ChAT-positive cells in the PPN expressed mCherry (Fig. 4*C*i). In the cerebellum, mCherry-expressing fibers were identified in the interpositus nuclei, within both the anterior and posterior interpositus (Fig. 4*C*), and also in the lateral cerebellar nuclei.

ChAT-Cre rats expressing either hM4Di or control mCherry were tested on the beam traversal task after cerebellar infusion of CNO or vehicle; example performance for a ChAT-hM4Di animal whose performance level was close to average is shown in Figure 4*G*. We identified statistically significant interactions between treatment and group for tail movements (using GLM assessing interactions between treatment and group while accounting for individual animal variability; *χ*^2^_(1)_ = 5.4; *p* = 0.02; Fig. 4*D*). The number of tail movements was significantly decreased following CNO infusion in ChAT-hM4Di (determined using post hoc simple effects analysis for the effect of treatment in the ChAT-hM4Di group; *χ*^2^_(1)_ = 5.67; *p* = 0.01) compared with CNO infusion in control rats. Similarly, statistically significant interactions between treatment and group were identified for foot placement score (GLM for treatment and group interactions; *χ*^2^_(1)_ = 6.5; *p* = 0.01; Fig. 4*E*). In the ChAT-hM4Di group, we found a significant decrease in the foot placement score following CNO infusion compared with vehicle (post hoc simple effects analysis for the treatment factor in the ChAT-hM4Di group; *χ*^2^_(1)_ = 7.9; *p* = 0.004). CNO infusion had no detectable effect on the control group (post hoc simple effects analysis for treatment factor in the control group) for tail movements (*χ*^2^_(1)_ = 0.1; *p* = 0.71) nor for foot placement score (*χ*^2^_(1)_ = 0.45; *p* = 0.49). No changes were noted in traversal time (*F*_(1, 10)_ = 0.64; *p* = 0.44; Fig. 4*F*). Together these results provide evidence that disrupting cholinergic PPN projections to the cerebellar nuclei improves beam traversal performance.

### Effects of pharmacologically enhancing cholinergic signaling

Our experiments show that inhibition of cholinergic PPN–cerebellar projections improves foot placement and balance during habitual performance of a beam traversal task in rats, indicating that a reduction in ACh in the interpositus cerebellar nuclei improved beam traversal performance. We next asked how an increase in ACh levels would impact task performance, i.e., to impair beam traversal.

**Figure 5.**
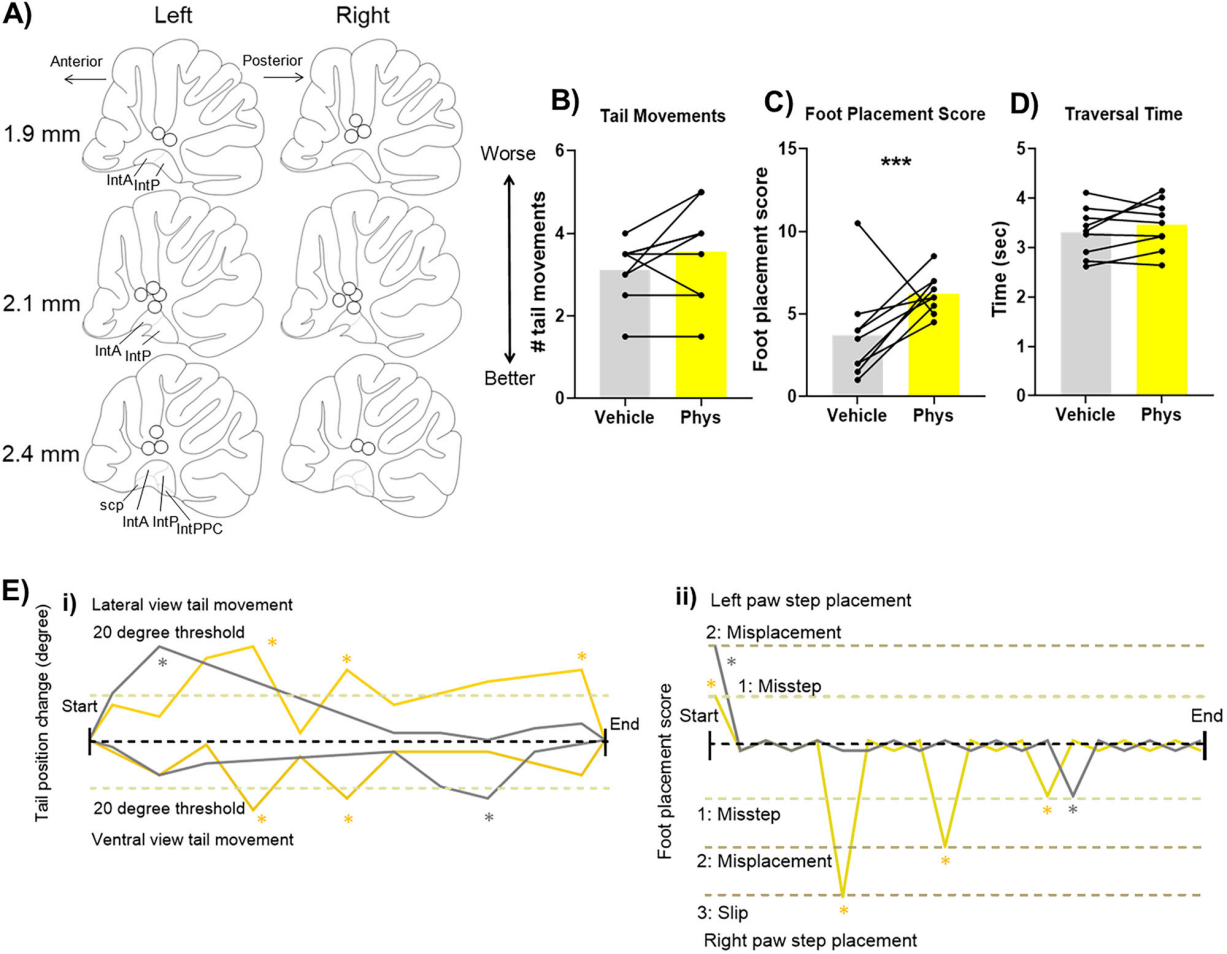
Pharmacologically enhancing cholinergic signaling impaired motor performance. ***A***, Cannulae implant location for *n* = 9 rats involved in the study. A, anterior; IntA, interpositus anterior; IntP, interpositus posterior; IntPPC, interpositus posterior parvicellular part; P, posterior; scp, superior cerebellar peduncle. ***B–D***, Infusion of the cholinesterase inhibitor physostigmine into the cerebellum resulted in a significant increase in (***C***) foot placement score, no change in the number of (***B***) tail movements, and no change in (***D***) traversal time (*n* = 9). Statistical analysis tested for differences with treatment. Stars indicate statistical significance: *** *p* < 0.001. ***E***, Example demonstrating the quantification of scored behavior in an example trial. Data example with vehicle infusion (gray traces) and physostigmine infusion (yellow traces). ***i***, Tail movement quantification for ventral and lateral view: any change >20° from the previous position was classified as a balance adjustment tail movement (indicated by gray stars for vehicle infusion and yellow stars for physostigmine infusion). ***ii***, The foot placement score across the trials showing both the left and right paw step placement: scores of each foot placement from the start of the trial to the end; gray stars for vehicle infusion and yellow stars for physostigmine infusion indicate the occurrence of missteps (partial misplacement), misplacements, and foot slips.

We used physostigmine, a cholinesterase inhibitor, to enhance endogenous ACh levels by delivering it locally in the interpositus nucleus (Fig. 5*A*; Pickford et al., 2024). Physostigmine significantly increased the foot placement score compared with vehicle (*χ*^2^_(1)_ = 22.2; *p* = 0.00002; *n* = 9 rats; Fig. 5*C*) but had no effect on tail movement (*χ*^2^_(1)_ = 1.1; *p* = 0.31; Fig. 5*B*) or traversal time (*t*_(8) _= 1.2; *p* = 0.26; Fig. 5*D*). Example tail movement and foot placement performance of a rat performing close to the group average is shown in Figure 5*E*. As hypothesized, we show that increasing ACh levels locally in the interpositus impairs motor performance by increasing foot placement errors on the beam traversal task. Together, these experiments suggest that decreasing levels of ACh in the cerebellar interpositus nucleus improves motor performance, while increasing ACh levels impairs motor performance.

### Effects of inhibiting specific nicotinic or muscarinic ACh receptors

Given that there are both nicotinic and muscarinic receptors in the cerebellum, we sought to determine the receptors responsible for the cholinergic effect on beam traversal performance. Thus, we infused nicotinic and subtype-specific muscarinic receptor antagonists into interpositus nuclei.

**Figure 6.**
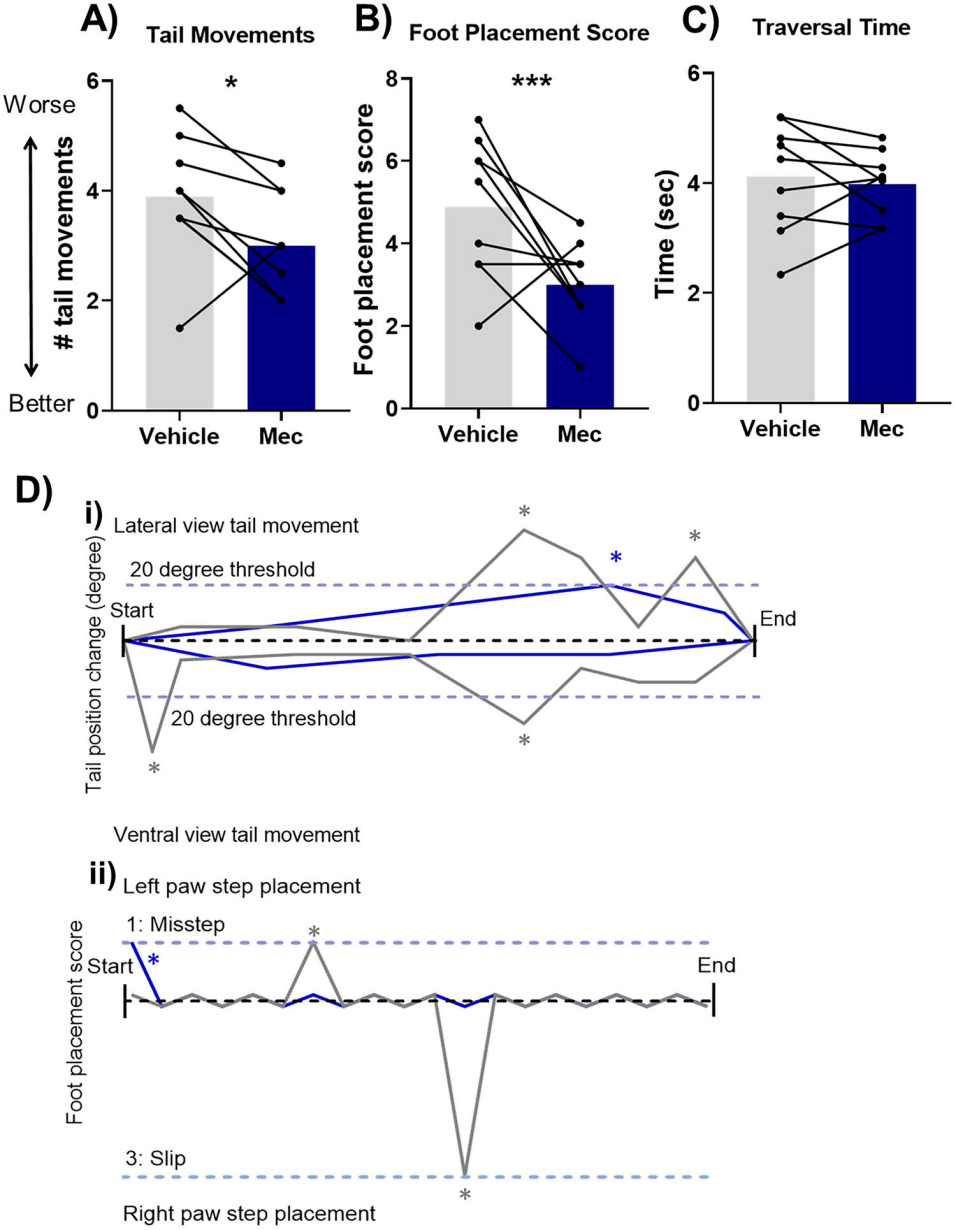
Inhibiting ACh nicotinic receptors improved motor performance. ***A–C***, Infusion of the nicotinic receptor antagonist mecamylamine into the cerebellum resulted in a significant reduction in the number of (***A***) tail movements and (***B***) foot placement score, with no change in (***C***) traversal time. Statistical analysis tested for differences with treatment. Stars indicate statistical significance: *p < 0.05; ***p < 0.001. ***D***, Example demonstrating the quantification of scored behavior in an example trial. Data example with vehicle infusion (gray traces) and mecamylamine infusion (blue traces). ***i***, Tail movement quantification for ventral and lateral view: any change >20° from the previous position was classified as a balance adjustment tail movement (indicated by gray stars for vehicle infusion and blue stars for mecamylamine infusion). ***ii***, The foot placement score across the trials showing both the left and right paw step placement: scores of each foot placement from the start of the trial to the end; gray stars for vehicle infusion and blue stars for mecamylamine infusion indicate the occurrence of missteps (partial misplacement), misplacements, and foot slips.

We first infused nicotinic receptor antagonist mecamylamine in the interpositus nuclei during beam traversal (same cannulae placements as shown in Fig. 5*A*). Mecamylamine significantly decreased the number of tail movements (χ^2^_(1)_ = 4.1; *p* = 0.04; Fig. 6*A*) and significantly reduced the foot placement score (χ^2^_(1)_ = 16; *p* = 0.0006; Fig. 6*B*). We identified no statistically significant difference for traversal time (*t*_(8)_ = 0.5; *p* = 0.61; Fig. 6*C*). Example tail movement and foot placement performance of an average-performing rat is shown in Figure 6*D*.

**Figure 7.**
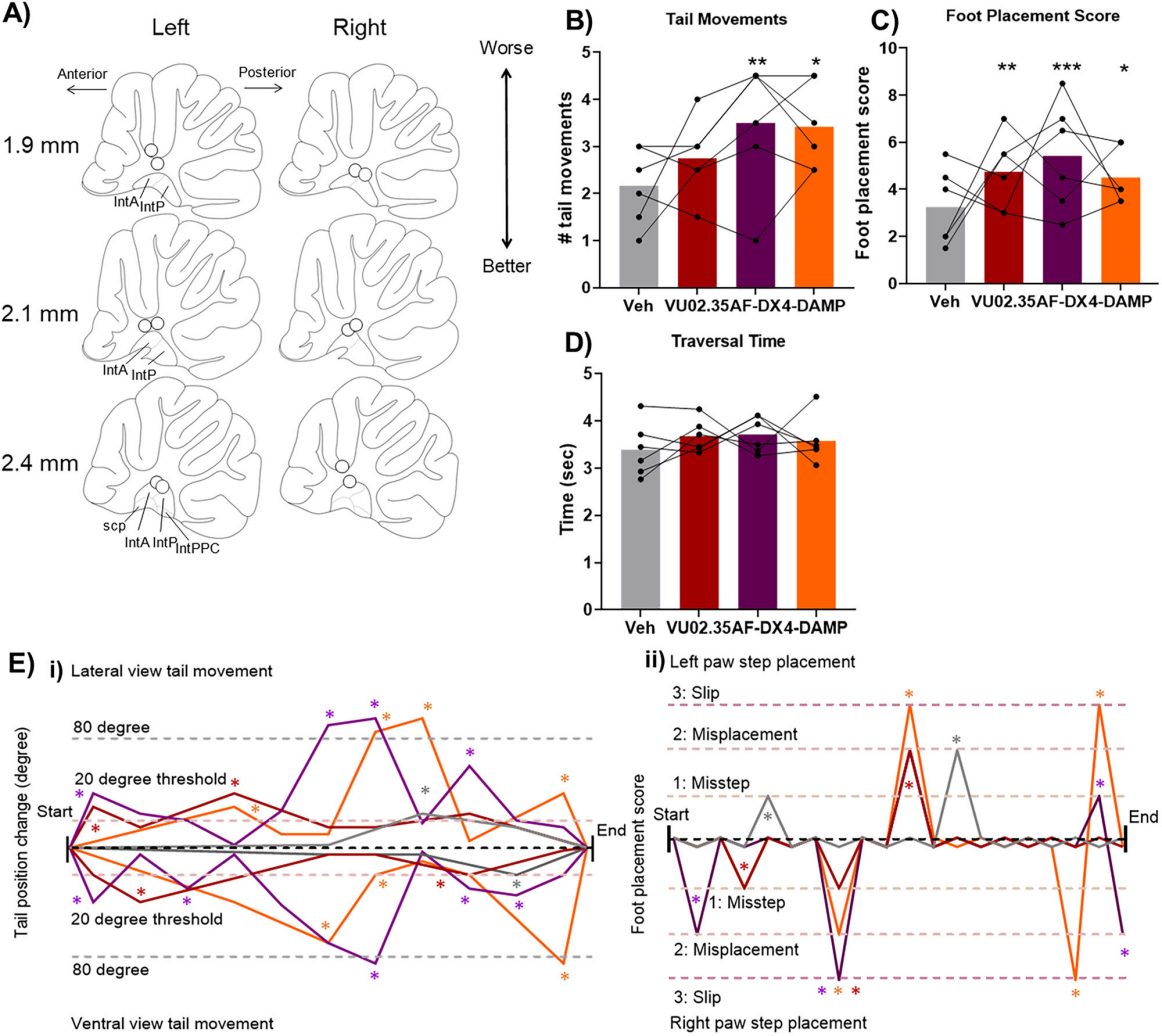
Infusion of specific muscarinic ACh receptor antagonists impaired motor performance. ***A***, Cannula implant location for *n* = 6 rats involved in the study. A, anterior; IntA, interpositus anterior; IntP, interpositus posterior; IntPPC, interpositus posterior parvicellular part; P, posterior; scp, superior cerebellar peduncle. ***B–D***, Infusion of muscarinic receptor antagonists into the cerebellum results in a significant increase in the number of (***B***) tail movements and (***C*)** foot placement score, with no change in (***D*)** traversal time. M1 muscarinic receptor antagonist (VU02.35) significantly increased the foot placement score. M2 muscarinic receptor antagonist (AF-DX) significantly increased the number of tail movements and foot placement score. M3 muscarinic receptor antagonist (4-DAMP) increased tail movements and foot placement score. Statistical differences based on the treatment factor are indicated by the star. Post hoc pairwise comparisons of individual treatments with vehicle are indicated by the stars above each bar. Stars indicate statistical significance: *p < 0.05; **p < 0.01; ***p < 0.001. ***E***, Example demonstrating the quantification of scored behavior in an example trial. Data example with vehicle infusion (gray traces) and muscarinic receptor antagonists infusions (red, purple, and orange traces for M1, M2, and M3 receptor antagonists, respectively). ***i***, Tail movement quantification for the ventral and lateral view: any change >20° from the previous position was classified as a balance adjustment tail movement (indicated by gray stars for vehicle infusion and red stars for VU0255035, purple stars for AF-DX116, and orange stars for 4-DAMP infusions). ***ii***, The foot placement score across the trials showing both the left and right paw step placement: scores of each foot placement from the start of the trial to the end; gray stars for vehicle infusion and red stars for VU0255035, purple stars for AF-DX116, and orange stars for 4-DAMP infusions indicate the occurrence of missteps (partial misplacement), misplacements, and foot slips.

We infused a separate group of rats with subtype-specific muscarinic receptor antagonists (*n* = 6 rats; cannula placement shown in Fig. 7*A*). We found statistically significant differences in the number of tail movements with the different treatments (*χ*^2^_(3)_ = 9.4; *p* = 0.02; Fig. 7*B*). Post hoc pairwise comparisons found a statistically significant increase in tail movements for the infusion of the M2 antagonist (AF-DX116, *χ*^2^_(1)_ = 7.4; *p* = 0.006) and M3 antagonist (4-DAMP, *χ*^2^_(1)_ = 6.6; *p* = 0.01) but not for the M1 antagonist (VU0255035, *χ*^2^_(1)_ = 1.16; *p* = 0.19). The different treatments also led to a statistically significant difference in foot placement score (*χ*^2^_(3)_ = 14.3; *p* = 0.006; Fig. 7*C*). In this case, pairwise comparisons found a significant increase for all subtype-specific muscarinic receptor antagonists: M1 (VU0255035, *χ*^2^_(1)_ = 6.6; *p* = 0.009), M2 (AF-DX116, *χ*^2^_(1)_ = 12.8; *p* = 0.0003), and M3 (4-DAMP, *χ*^2^_(1)_ = 4.8; *p* = 0.02). Example tail movement and foot placement performance across the different treatments is shown in Figure 7*E*. We identified no significant changes in traversal time (*F*_(1.9,9.7)_ = 0.7; *p* = 0.5; Fig. 7*D*).

We found that inhibiting nicotinic receptors in the interpositus nuclei improved accuracy of movement indicated by fewer tail movements and lower foot placement score. At the same time, we found that disrupting cerebellar muscarinic signaling impairs balance and limb coordination on the beam traversal task in rats. Thus, our results indicate that the effect of cholinergic PPN projections to the cerebellar nuclei are primarily exerted through modulation of nicotinic receptors, as has been suggested by in vivo electrophysiology studies (Vitale et al., 2016).

### Cellular effects of cholinergic receptor activation

Our behavioral experiments found opposing effects on motor performance with inhibition or enhancement of cholinergic signaling within the interpositus. Our next experiments therefore set out to understand the cellular effects of altering ACh levels in the cerebellar nuclei. On average, the cerebellar interpositus cells recorded from *N* = 30 cells from *n* = 30 rats had a large soma (305.1 ± 29 µm^2^ surface area, consistent with a cell diameter or 23.9 ± 1 µm), with an average 5.3 ± 0.3 primary neurites and ∼2.4 ± 0.3 secondary branch points per neurite. Based on these morphological features, plus their spontaneous firing rate (∼5 ± 0.5 Hz) and AP half-width (∼1 ± 0.1 ms), we identified these neurons as putative glutamatergic projection neurons (Fig. S2; Uusisaari and Knopfel, 2011; Kebschull et al., 2024).

**Figure 8.**
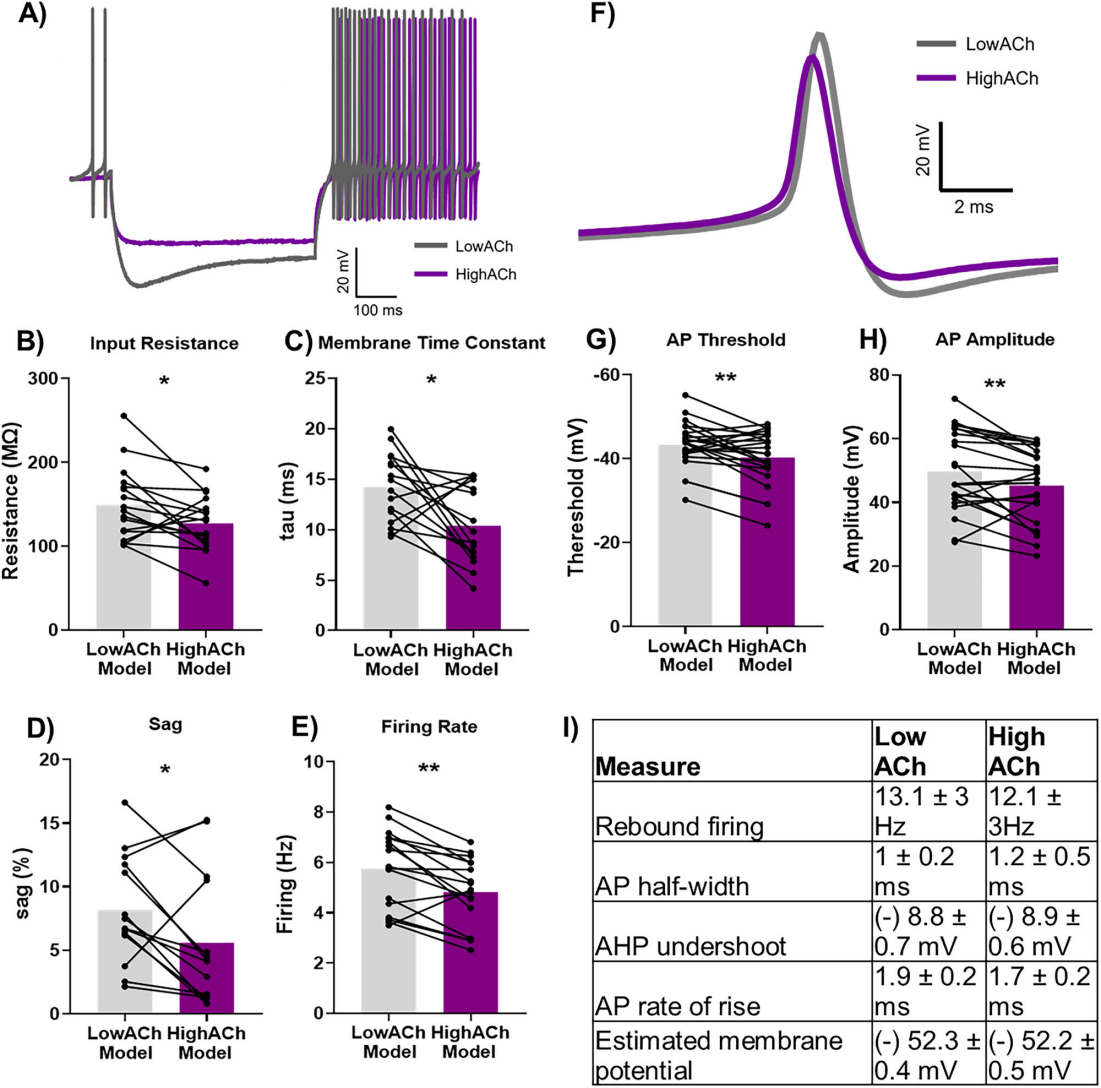
In vitro model of low and high ACh conditions found changes in cell excitability with cholinergic receptor activation. ***A***, To determine the intrinsic properties of the membrane, cells were injected with a square hyperpolarizing current step (−300 mV). Membrane input resistance (Ri), membrane time constant (tau), and sag were calculated post hoc. Example response following hyperpolarizing current injection under low ACh (gray) and high ACh (purple) conditions. ***B–E***, Cholinergic receptor activation via bath application of the cholinergic agonist carbachol (High ACh Model) significantly reduced (***B***) input resistance, (***C***) membrane time constant, (***D***) sag, and (***E***) firing rate of neurons in the interpositus cerebellar nuclei in vitro, compared with baseline (Low ACh Model). ***F–H***, Carbachol application changed the (***F***) waveform of APs. The (***G***) AP threshold was significantly increased and (***H***) AP amplitude was significantly decreased in the High ACh Model compared with the Low Ach Model, indicating that cells need to be more depolarized in order to fire an AP. ***I***, Properties unchanged by high cholinergic conditions: rebound firing (firing rate posthyperpolarizing current injection), AP half-width (width of AP halfway between threshold and peak), AHP undershoot (hyperpolarization post AP peak, measured relative to threshold), AP rate of rise (time from threshold to peak), estimated membrane potential (membrane potential measured between APs). For all graphs, the analysis measured changes in electrophysiological properties with different treatment conditions. Stars indicate statistical significance: *p < 0.05; **p < 0.01.

We extracted neuronal intrinsic properties via hyperpolarization current injection at baseline and following carbachol perfusion (Fig. 8*A*). We assumed a low level of ACh in the slices under baseline conditions as all cholinergic sources were severed during the slicing process. In accordance with this, we have previously shown that antagonizing muscarinic receptors under such conditions had no effect on the firing rate, membrane potential, or synaptic responses of interpositus neurons in slices (Pickford et al., 2019). We therefore compared the “Low ACh” baseline condition with that in the presence of carbachol (“High ACh”). Cholinergic receptor activation with carbachol significantly decreased input resistance (*t*_(16)_ = 2.4; *p* = 0.03; Fig. 8*B*), membrane time constant (*t*_(15)_ = 2.9; *p* = 0.01; Fig. 8*C*), sag (*t*_(13)_ = 2.3; *p* = 0.03; Fig. 8*D*), and average spontaneous firing rate (*t*_(16)_ = 3.9; *p* < 0.01; Fig. 8*E*), indicative of a decrease in cell activity (Meng et al., 2016). In addition, cholinergic receptor activation significantly increased AP firing threshold (*t*_(23)_ = 3.1; *p* = 0.004; Fig. 8*G*) and decreased AP peak amplitude (*t*_(23)_ = 2.8; *p* = 0.01; Fig. 8*H*). We identified no detectable differences in rebound firing, AP half-width, AHP, AP rate of rise, nor estimated membrane potential following prolonged (10 min) carbachol application (Fig. 8*I*). However, there was a significant depolarization of membrane potential early in carbachol application (1–3 min) which returned to baseline levels by 10 min (Fig. S3*A*; F_(1.4,30.1)_ = 2.9; *p* = 0.08; multiple comparisons, low ACh vs start ACh *p* = 0.007; low ACh vs high ACh *p* = 0.99). The spontaneous firing rate followed a different pattern, showing an increase in firing the start of carbachol application (1–3 min) but a decrease at 10 min (Fig. S3*B*; *F*_(1.4,20)_ = 11.6; *p* = 0.001; multiple comparisons, low ACh vs start ACh *p* = 0.04; low ACh vs high ACh *p* = 0.001).

Taken together, these results suggest that enhanced cholinergic receptor activation in the cerebellar nuclei can reduce the excitability of interpositus nuclear neurons in vitro. This is consistent with our in vivo results, as they suggest that the deficit in motor control we observed following infusion of physostigmine into the interpositus nuclei was due to enhancement of ACh levels producing a decrease in neuronal excitability.

To test for effects on synaptic transmission, we also evoked IPSCs and EPSCs. Bath perfusion of carbachol significantly decreased the amplitude of both IPSCs and EPSCs (IPSCs, *t*_(3)_ = 8.9; *p* = 0.002; EPSCs, *t*_(3)_ = 3.8; *p* = 0.03; Fig. S4). By comparison, we found no statistically significant difference in paired-pulse ratios for IPSCs (*t*_(3)_ = 0.6; *p* = 0.58; from 0.99 ± 0.002 at baseline to 1.00 ± 0.004 after carbachol) nor EPSCs (*t*_(3)_ = 0.6; *p* = 0.55; from 1.01 ± 0.004 at baseline to 1.01 ± 0.002 after carbachol). In summary, these cellular results provide evidence that cholinergic signaling can reduce both the excitability of cerebellar nuclear neurons and their responsiveness to synaptic inputs.

## Discussion

We have shown that skilled motor performance, assessed by a beam traversal task, can be bidirectionally modulated by manipulations of cholinergic signaling in the cerebellum. Chemogenetically inhibiting PPN–interpositus nuclei projections or antagonizing nicotinic receptors in the interpositus nuclei reduced the number of compensatory tail movements and foot placement errors during beam traversal, consistent with improved balance and task performance. Conversely, enhancing ACh levels pharmacologically using a cholinesterase inhibitor or antagonizing muscarinic receptors increased the number of foot placement errors and compensatory tail movements, consistent with impaired balance and motor performance. At the cellular level, we show that prolonged pharmacological activation of ACh receptors in vitro reduced the excitability of interpositus neurons, indicating that increased levels of ACh may attenuate cerebellar output. Taken together, these results show that decreasing ACh levels in the interpositus nuclei improves motor skills required for beam traversal, whereas increasing ACh leads to an impairment of these skills, potentially through a reduction in cerebellar nuclei output. The effects of our manipulations appear to be specific to skilled motor control as inhibiting the PPN–cerebellar nuclei pathway had no effect on general locomotor activity during open-field exploration.

### Behavioral implications

The interpositus nuclei are important for coordinating limb movements, including those involved in locomotion, and neuronal activity is step cycle dependent (Armstrong and Edgley, 1984; Apps and Garwicz, 2005; Sánchez-Campusano et al., 2007). In rodents, tail movements are closely related to the step cycle; specifically, larger tail momentum is associated with lower body momentum during the swing phase of the step cycle (Lacava et al., 2024). We chose a beam traversal task to test stepping accuracy under challenging conditions, which also allowed us to measure compensatory tail movements which may not be needed in conditions without additional demands for balance.

We interpreted fewer tail movements as improved performance because there is a reduced need to regain balance. In support of this, the number of compensatory tail movements was increased in a rat model of olivocerebellar ataxia (Wecker et al., 2013). The same study showed that systemic administration of nicotinic receptor agonists improved measures of balance and gait in the model of ataxia (Wecker et al., 2013), highlighting the therapeutic potential of targeting the cholinergic system for motor disorders. However, the improvement in motor control with nicotinic agonists reported by Wecker et al. (2013) contrasts with our finding that infusion of a nicotinic receptor antagonist into the cerebellar nuclei improves motor performance. This difference could be explained by a local versus systemic administration method or the possibility that the cholinergic system may make different contributions to motor control in physiological and pathophysiological states. Whether an improvement or impairment in behavior occurs may depend on the baseline ACh level.

Our results show that increased ACh in the interpositus nuclei is detrimental to motor performance and that cholinergic receptor activation reduced the excitability of cerebellar nuclear neurons in vitro. The latter finding is supported by our previous in vitro work in juvenile rats which showed that a cholinergic agonist produced an initial rapid muscarinic receptor-dependent increase in the firing rate of cerebellar nuclear neurons, which was subsequently attenuated within minutes while the agonist was still present (Pickford et al., 2019). Relevant to beam traversal, the interpositus nuclei modulate the accuracy of fine limb movements. In the reach-to-grasp task, accuracy of limb movement is controlled by an increase in the firing rate in the anterior interpositus nuclei, resulting in a deceleration of the paw as it approaches the target (Becker and Person, 2019). Therefore, prolonged or excessive cholinergic receptor activation, as seen in our experiments, could impair limb placement accuracy by decreasing cerebellar neuronal excitability.

### Cellular effects

Our work has explored the behavioral effects of subtype-specific cholinergic receptor inhibition, and in vitro experiments showed that prolonged ACh receptor activation decreased cellular excitability. Considering that the cerebellum has (1) high levels of AChE, the enzyme which degrades ACh (Friede, 1964; Silver, 1967); (2) high expression of M2 muscarinic receptors, which inhibit ACh release (Jaarsma et al., 1997); and (3) high affinity nicotinic receptors which desensitize within milliseconds (Ochoa et al., 1989), we propose that the cerebellum has developed to perform optimally with low ACh levels.

**Figure 9.**
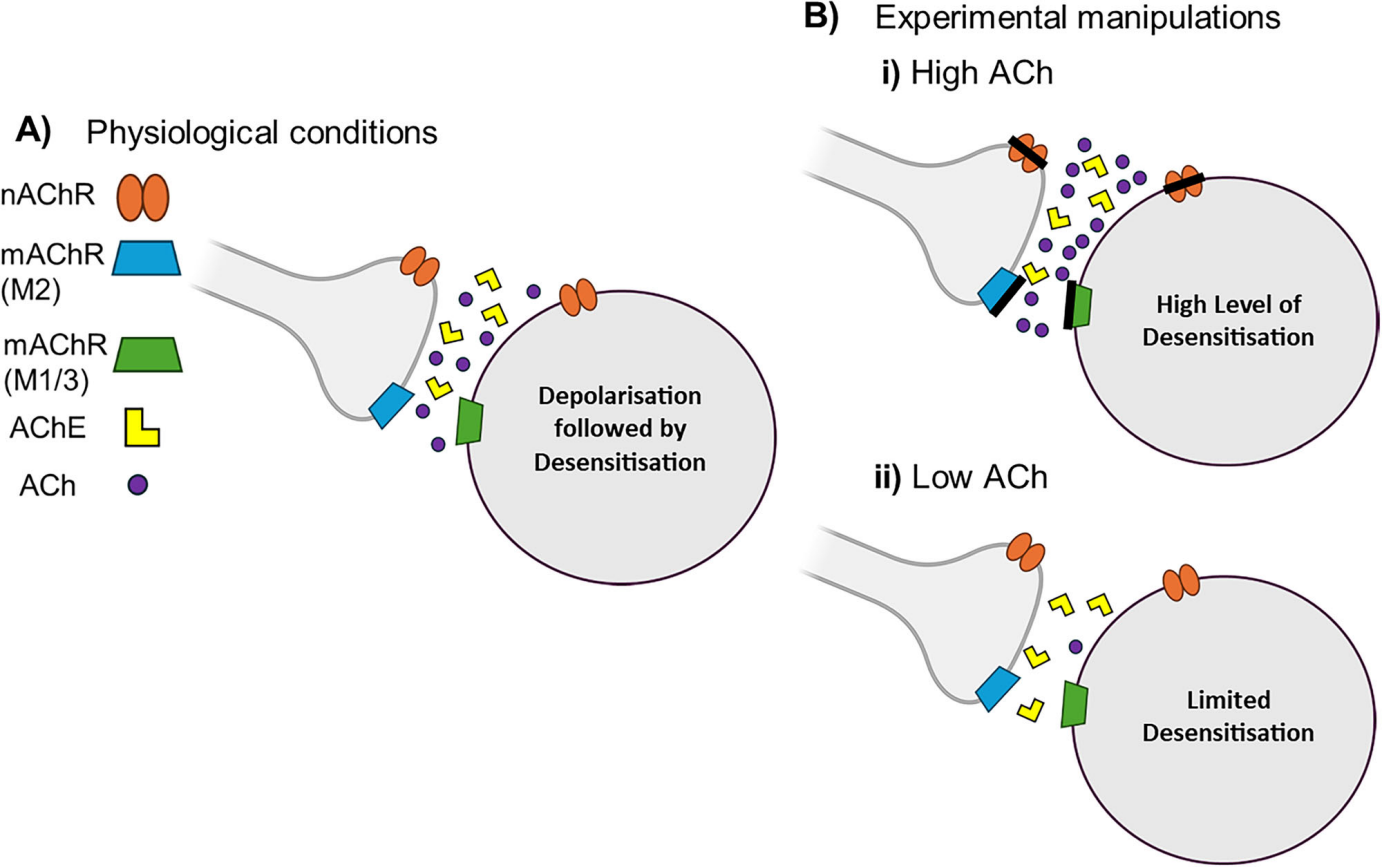
Theory of cholinergic signaling in the cerebellar nuclei: proposed mechanism under different conditions. ***A***, ACh activates presynaptic and postsynaptic nAChR and mAChR: postsynaptic receptors are generally excitatory (nAChR and Gq mAChR), while presynaptic receptors are both excitatory (nAChR) and inhibitory (Gi mAChR). ACh release leads to an excitatory response (mediated by fast nAChR and slow mAChR), and release is sustained in the initial stages. Generally, ACh release lasts a few seconds (Neyhart et al., 2024); AChE clears ACh in milliseconds (Massoulié et al., 1993), while it takes nAChR tens of milliseconds to desensitize (Ochoa et al., 1989); mAChR remain active for minutes (Rajagopal and Shenoy, 2018). Thus, by the end of release when AChE degrades ACh and terminates transmission, the net effect is cellular depolarization with some degree of nAChR desensitization. ***B***, Experimental conditions: ***i***, in high cholinergic conditions (via inhibiting cholinesterase and muscarinic receptor antagonization) high levels of ACh desensitize nAChR, and prolonged activation leads to mAChR deactivation, desensitization, and/or internalization. Antagonizing M2 muscarinic receptors may increase ACh release by inhibiting presynaptic autoreceptors on cholinergic terminals, thereby replicating the effects of physostigmine. Increased ACh levels through either of these routes may lead to greater desensitization of postsynaptic muscarinic receptors (M1 and M3 subtypes) or lead to an increase in the muscarinic depression of excitability. ***ii***, In low cholinergic conditions (PPN chemogenetic inhibition and nicotinic receptor antagonization), the low levels of ACh activate synaptic cholinergic receptors and induce depolarization by activating a limited number of receptors. Mecamylamine administration could therefore (1) reduce ACh release by blocking presynaptic nicotinic receptors and/or (2) replicate the reduced postsynaptic nicotinic receptor activation that occurs when the PPN–cerebellar nuclei input is inhibited. Lower ACh levels may be rapidly cleared by AChE, allowing a brief increase in postsynaptic cell excitability and having a positive effect on performance.

Under physiological conditions during motor behavior, it is possible that ACh release is ongoing, leading to rapid cerebellar nuclei neuronal firing driven initially by nicotinic receptors and then maintained by muscarinic Gq-coupled receptors (Fig. 9*A*). The cerebellar nuclei express nicotinic receptors, most likely composed of α4 and β2 subunits (Jaarsma et al., 1997). Activation of these receptors induces membrane depolarization through a rapid influx of ions, which can subsequently activate Ca^2+^ channels (e.g., L-type) and produce brief depolarizing events (Letz et al., 1997; De Filippi et al., 2001). These events are transient because nicotinic receptors typically desensitize within tens to hundreds of milliseconds (Ochoa et al., 1989; Yu et al., 2009). While the precise timescale of ACh release in the cerebellar nuclei remains to be determined, findings from the visual cortex indicate that ACh release occurs over ∼1 s ahead of motor onset, with decay lasting up to 5 s (Neyhart et al., 2024). Thus, physiological instances of repeated or prolonged ACh release are likely to result in nicotinic receptor desensitization (Fig. 9*A*), as are conditions involving pharmacological increase of ACh, such as those in our experiments (Fig. 9*B*).

Gq-coupled M1 and M3 muscarinic receptors are typically postsynaptic (Groleau et al., 2015; Oda et al., 2018) and have been identified in the cerebellum (Rinaldo and Hansel, 2013; Pickford et al., 2019). Activation of postsynaptic muscarinic receptors triggers a signaling cascade that enhances membrane excitability via multiple mechanisms, including (1) modulation of HCN channels, which regulate resting membrane potential and responsiveness to synaptic input (Bronson and Kalluri, 2023; Beaver and Evans, 2025); (2) reduction of potassium leak currents, which act to stabilize resting membrane potential (their reduction therefore leads to depolarization and increased excitability; Vanner et al., 1993; Goldstein et al., 2001); and (3) voltage-gated potassium channels can be suppressed via Gq-coupled pathways which reduce the M-current, further enhancing excitability (Brown and Passmore, 2009).

Muscarinic receptors desensitize following prolonged activation, over a longer time course than nicotinic receptors (Schmidt et al., 1995; Rajagopal and Shenoy, 2018). This supports our current findings which show that prolonged exposure to a cholinergic agonist decreased excitability of interpositus nuclei neurons, suggesting that ACh receptors in the cerebellar nuclei become desensitized over time (Rajagopal and Shenoy, 2018). Increasing ACh levels in the cerebellum may therefore lead to a higher rate of desensitization (Fig. 9*B*i).

Approximately 90% of muscarinic receptors in the cerebellum are of the M2 subtype (Jaarsma et al., 1997). M2 receptors are predominantly presynaptic and act as inhibitory autoreceptors to regulate ACh release (Zhang et al., 2002). Antagonizing M2 muscarinic receptors therefore increases ACh release (Zhang et al., 2002). This may explain our finding that both muscarinic receptor antagonists and physostigmine produce a behavioral impairment, supporting the idea that elevated cerebellar ACh levels are detrimental to motor behavior. Increased ACh may lead to enhanced desensitization of postsynaptic muscarinic receptors and/or lead to an increase in the muscarinic depression of excitability.

We propose that lower ACh levels, as in our chemogenetic experiments, may enhance interpositus neuronal excitability (and therefore motor performance) by restricting receptor activation and desensitization and/or the limiting the decrease in excitability seen in our in vitro experiments (Fig. 9*Bii*). Mecamylamine may mimic these effects by blocking presynaptic nicotinic receptors, thereby decreasing ACh release, and/or by reducing postsynaptic nicotinic receptor activation, which would shorten the duration of depolarization.

### Concluding comments

Evidence from disease states has shown that optimal cholinergic tone is required for motor function, as too much (e.g., in the basal ganglia in Parkinson’s disease; Aosaki et al., 2010) or too little (e.g., PPN degeneration; Chambers et al., 2020) ACh can result in motor deficits (Maurice et al., 2015; Pagano et al., 2015; Rizzi and Tan, 2017; Huang et al., 2022; Matityahu et al., 2023). Our findings extend this to the physiological state and specifically to the role of ACh in motor functions mediated by the cerebellum. The ability to positively impact performance from the physiological state could inform the development of performance-enhancing drugs, together with providing considerations for the side effects of cholinergic drugs which are used both clinically and recreationally.

We have studied the effects of cholinergic signaling on performance in well-trained animals. In the hippocampus, high levels of ACh are important for memory encoding but not recall (Hasselmo, 2006), so it is possible that different levels of ACh in the cerebellum are optimal for habitual versus novice performance. Future studies could test this possibility. In addition to its motor roles, it is likely that cholinergic projections to the cerebellum are involved in a host of its other functions including cognitive, emotional, and homeostatic processes. The ACh level may be balanced to serve multiple functions and be adequate for each rather than optimal for a single function, which may be why we were able to improve performance on a demanding locomotion task from the physiological state.
